# Simultaneous heavy metal removal and anthracene biodegradation by the oleaginous bacteria *Rhodococcus opacus*

**DOI:** 10.1007/s13205-016-0597-1

**Published:** 2017-04-24

**Authors:** Lalit Goswami, N. Arul Manikandan, Kannan Pakshirajan, G. Pugazhenthi

**Affiliations:** 1 Center for the Environment, Indian Institute Technology Guwahati, Guwahati, Assam 781039 India; 2 Department of Chemical Engineering, Indian Institute Technology Guwahati, Guwahati, Assam 781039 India; 3 Department of Biosciences and Bioengineering, Indian Institute Technology Guwahati, Guwahati, Assam 781039 India

**Keywords:** Biodegradation, Anthracene, Heavy metals, *Rhodococcus opacus*, Lipid accumulation

## Abstract

This study investigated simultaneous heavy metals removal and anthracene biodegradation by *Rhodococcus opacus* at different initial anthracene concentrations in the range 50–200 mg L^−1^. The heavy metals tested were Fe(III), Cu(II), Zn(II), Cd(II), Ni(II), and Pb(II) at 10 mg L^−1^ initial concentration: The organism was found to be well capable of removing the heavy metals along with high anthracene biodegradation efficiency. However, anthracene biodegradation rate by the organism was reduced due to these heavy metals. In addition, the heavy metals effect on *R. opacus* biomass growth followed the order: Cd > Ni > Pb > Cu > Zn > Fe. The total time to anthracene biodegradation increased from 144 to 216 h in the presence of Fe, Zn, Cu, or Pb, and it was up to 240 h in the presence of Cd or Ni. Compared with 70.2% (w/w) lipid accumulation by the bacterium in the absence of these heavy metals, a significant decline in the same was observed in the presence of the different heavy metals. These values were 41.2, 44.1, 52.1, 54.1, 58.6, and 63.1% (w/w) for Cd, Ni, Pb, Cu, Zn, and Fe, respectively. Field emission scanning electron microscopy integrated with energy dispersive X-ray spectroscopy and transmission electron microscopy of the biomass grown in the presence and absence of these heavy metals further confirmed a change in morphology of the bacterium due to the heavy metals. Fourier transmission infrared spectroscopy spectra of the biomass obtained during its growth in the presence and absence of the heavy metals confirmed the involvement of N–H, C–H bend, –CH_2_–(C=O), C–N stretch, C–H and O–H bending, and –C–Cl groups on the biomass for heavy metal uptake by the bacterium.

## Introduction

Release of different pollutants into the environment has increased noticeably, thereby diminishing the environment quality to frightening levels (Kiran et al. 2016; Roy et al. 2015). These pollutants are known to affect both the living and non-living assets of the biosphere via numerous routes (Ye et al. 2013). Among the different environmental pollutants, polycyclic aromatic hydrocarbons (PAHs) and heavy metals (HMs) are the most important because of their persistence, recalcitrance, abundance, and toxicity in the environment (Arul Manikandan et al. 2016; Titaley et al. 2016; Gopi Kiran et al. 2015; Samanta et al. 2002).

Heavy metals, such as copper, zinc, and iron, despite being toxic at a high concentration, they are essential for the growth of microorganisms in trace amounts (Baldrian et al. 2000; Hiroki et al. 1985). PAHs are toxic, mutagenic, carcinogenic, teratogenic, and persistent in the environment mainly due to their hydrophobic and water insoluble properties. Some PAHs are known to be a human skin photosensitizer, mild allergen, whereas some other PAHs are reported to cause haemolytic anaemia and nephrotoxicity (Bücker et al. 1979). Heavy metals are widely used in metal finishing, leather tanning, electroplating, nuclear power plant, and textile industries, whereas PAHs are ubiquitously found at wood preservation plants, industrial sites associated with petroleum and coal tar manufacturing, gas plants, runoff from asphalt pavement, and combustion processes (Kiran et al. 2016; Hadibarata and Kristanti 2012; Yap et al. 2011; Mahanty et al. 2008; Samanta et al. 2002). Therefore, industrial effluent containing these contaminants when released from the main stream affects each other’s biodeterioration and biotransformation by microorganism. For instance, heavy metals, including cadmium (Cd), lead (Pb), and nickel (Ni), are shown to adversely affect biodegradation of organic contaminants in the environment (Atagana 2006; Riis et al. 2002; Sokhn et al. 2001).

Remedial technologies, viz., volatilization, chemical oxidation, adsorption, photo-oxidation, and bioaccumulation, have been investigated for treating PAHs contaminated systems (Haritash and Kaushik 2009). Microbial biodegradation and biotransformation is proving to be more successful than these conventional techniques considering such issues as safety, cost, degradation efficiency, etc. Bacterial species, including *Bacillus, Burkholderia, Mycobacterium, Pseudomonas, Rhodococcus,* and *Sphingomonas,* are known to degrade/mineralize different PAHs into simpler non-hazardous metabolites. Organisms belonging to the actinomycete group are well known for PAHs utilization along with triacylglyceride (TAG) accumulating capability, which can be further transesterified for potential biodiesel application. *Rhodococcus, Nocardia, Mycobacterium,* and *Streptomyces sp.* are few of them (Kumar et al. 2015; Kurosawa et al. 2010; Mehta and Chavan 2009).

In general, PAHs biodegradation using bacteria, algae, and fungi have widely been reported in the literature (Sasek et al. 2003; Daane et al. 2002; Alexander 1999; Cerniglia and Heitkamp 1989). But till date, there are no studies which were found in the literature on simultaneous uptake of heavy metals and PAH biodegradation. This is very important from the standpoint of designing biological processes for treating a variety of pollutants in mixture. Therefore, this study evaluated the simultaneous heavy metal removal and anthracene biodegradation by *R. opacus* with a view to assess the feasibility of remediating aqueous environment contaminated with both PAH and heavy metals. Anthracene, a tri-ring PAH, is often utilized in dye and insecticide manufacturing and material coating industries. Uptake of anthracene via numerous routes in the body imposes toxicity to the skin, blood, intestine, and the lymphatic system, thus leading to tumors, edema, itching, and upsurge of fluids in tissues (Wieczorek et al. 2015). The heavy metals tested for this removal by *R. opacus* were Fe(III), Cu(II), Zn(II), Cd(II), Ni(II), and Pb(II). Furthermore, the bacterial morphology and accumulated lipid globules during the bio-removal process were examined employing field emission scanning electron microscopy (FESEM) equipped with energy dispersive spectroscopy (EDX) and transmission electronic microscopy (TEM). Fourier transform infrared spectroscopy (FTIR) analysis was deployed to identify heavy metal-binding functional groups on the biomass for their removal.

## Materials and methods

### Microorganism and culture conditions

The bacterial strain (*Rhodococcus opacus* DSM 43205) utilized in this study was obtained from Microbial Type Cell Culture (MTCC, Chandigarh, India). The culture was primarily maintained by sub-culturing on Luria–Bertani (LB) agar plates at 4 °C and further preserved in 20% (v/v) glycerol at −80 °C. For anthracene biodegradation, Bushnell-Hass minimal salt medium (BHMSM) (pH 7) was utilized (Lee and Cho 2009). The bacterial growth conditions were 30 °C temperature and 120 rpm agitation speed.

### Chemicals and reagents

Anthracene (99% pure) was obtained from Himedia (Mumbai, India) with purity greater than 99%. Analytical grade chemicals for preparing the bacterial growth media were procured from either Merck (Mumbai, India) or Himedia (Mumbai, India). All the chemicals except solvent used in this study for preparing the bacterial growth media and for its cultivation were of analytical grade and obtained from Himedia (Mumbai, India). HPLC grade dichloromethane (DCM) and acetonitrile were purchased from Spectrochem (Mumbai, India). Individual metal stock solutions of Fe(III), Cu(II), Zn(II), Cd(II), Ni(II), and Pb(II) of concentration 1000 mg L^−1^ each were prepared using FeCl_3_, CuCl_2_·2H_2_O, ZnCl_2_, Cd(NO_3_)_2_, NiCl_2_·6H_2_O, and PbNO_3_, respectively.

### Experiment setup

Seed culture of the bacterial strain *Rhodococcus opacus* was prepared using BHMSM containing 20 mg L^−1^ of anthracene for studying the PAH biodegradation. For inoculum preparation, 1 mL of the culture obtained at its mid logarithmic growth phase was centrifuged (8000*g*, 10 min) to get pellets of intact bacterial cells. Following resuspension of the intact cells in BHMSM, 5% (v/v) of these bacterial cells were inoculated into 250 mL Erlenmeyer flask containing 93 ml of BHMSM and different initial anthracene concentrations in the range 50–200 mg L^−1^ as the sole carbon and energy source. The final volume was made up to 100 mL by adding individual heavy metal stock solution to achieve an initial concentration of 10 mg L^−1^. The initial concentration used in this study was chosen based on a literature study by Kiran et al. (2016) and Pavasant et al. (2006) for heavy metals removal using microalgae and anaerobic sludge, respectively. Ten percentage of DCM was then added to dissolve the PAH. Flasks with no inoculum, but with the heavy metals, anthracene, and BHMSM were used as control in these experiments. The flasks were placed in an orbital incubator shaker set at 120 rpm and 30 °C. Sample aliquots were taken at regular time intervals for quantitative analysis of *R. opacus* biomass, lipids, residual anthracene, and heavy metals.

## Analytical methods

### Analysis of anthracene and heavy metals

Anthracene concentration in the samples was determined by high-pressure liquid chromatography (HPLC), (Varian Prostar 210, The Netherlands). A standard solution of anthracene was prepared using pure dichloromethane (HPLC grade). C-18 column Thermo hypersil™ was utilized (100 × 4.6 mm) with an injection volume of 20 µL. Two mobile phases were used in the analysis: Milli Q water and acetonitrile in the ratio 70:30 with a constant flow rate of 1 mL min^−1^. The eluted compounds were detected at 254 nm and the retention time was compared with that of a known standard. The following Eq. (1) was used to determine the % anthracene degradation:

1 $${\text{Anthracene}}\,{\text{biodegradation}}\,(\% )\, = \,\left[ {\frac{{\left( {C_{0} - C_{\text{f}} } \right) - C_{\text{n}} }}{{C_{0} }}} \right] \times 100$$

where *C*_*o*_ is the initial anthracene concentration; *C*_f_ and *C*_n_ are the final concentrations in the test and control flasks, respectively.

Heavy metal concentration in the samples was analyzed by atomic absorption spectroscopy (Varian, AA240, The Netherlands) as per the American Public Health Association standards (APHA 2005).

### *Rhodococcus opacus* biomass growth

*Rhodococcus opacus* cell density was determined by measuring its absorbance at 660 nm using a UV–Vis spectrophotometer (Agilent Technologies, Cary 100 series, Singapore). The bacterial growth was determined based on a standard graph plotted between OD_660_ versus cell dry weight (CDW). The specific anthracene uptake rate and specific lipid accumulation rate by *R. opacus* were estimated as per the following Eqs. 2 and 3, respectively:

2 $${\text{Specific}}\,{\text{anthracene}}\,{\text{uptake}}\,{\text{rate}}\,(q_{\text{A}} )\, = \,\left( {\frac{1}{{C_{p} }}\frac{{dC_{p} }}{dt}} \right)$$

3 $${\text{Specific}}\,{\text{lipid}}\,{\text{accumulation}}\,{\text{rate}}\,(q_{\text{L}} )\, = \,\left( {\frac{1}{{C_{L} }}\frac{{dC_{L} }}{dt}} \right).$$

In the above equation, *q*_A_ is the specific anthracene uptake rate (h^−1^), *q*_L_ is the specific lipid accumulation rate (h^−1^), and *C*_p_ and *C*_L_ are the concentrations (mg L^−1^) of anthracene and lipid corresponding to time *t* (h), respectively.

### Total lipid content

Total lipid content in the biomass was determined by the standard chloroform and methanol extraction procedure with minor modifications (Kumar et al. 2015; Folch et al. 1957). 5 mL of sample aliquot was centrifuged (8000*g*, 10 min) and the resultant pellet was mixed with 2 mL solvent mixture containing chloroform and methanol (2:1 v/v), and kept over for 12 h. The mixture was then centrifuged (8000*g*, 15 min) and the lower phase containing lipid dissolved in chloroform was transferred into a pre-weighed centrifuge tube (W_1_). The extraction procedure was repeated twice. The centrifuge tube containing the total volume of the supernatant collected from each extraction was subjected to 60 °C in an oven to evaporate the solvents and then reweighed (W_2_). The amount of lipid in the samples was calculated from the difference between W_1_ and W_2_.

## Characterization of the bacterial biomass

### FESEM and TEM analyses

Changes in the bacterial surface morphology observed due to simultaneous uptake of heavy metals and anthracene were examined by field emission scanning electron microscopy (FESEM) (Zeiss, Sigma, Germany). The bacterium grown in the presence of both anthracene and Cu as the heavy metal was used for this analysis. 1 mL of the bacterial culture was centrifuged (10,000*g*, 10 min) and washed twice with sterile milliQ water. The pellet obtained was diluted ten times with milliQ water and vortexed. Unit drop of this sample was mounted on aluminum stubs over double-sided carbon tape and dried overnight at 30 °C prior to the analysis. After drying, the sample was coated with thin gold layer by sputter coater before FESEM observation. The spectra obtained were compared with the biomass grown in the absence of anthracene and heavy metals.

For determining the size of the accumulated lipid by *R. opacus*, transmission electron microscopy (TEM) (JEOL, JEM2100, Japan) at 200 kV was carried out. Single drop of the sample, prepared as mentioned above under FESEM analysis, was casted on copper grid coated with carbon (Tedpell, USA) and dried overnight at 30 °C before the analysis.

### FTIR and FESEM-EDX analysis

Infrared spectroscopy of the biomass grown in the presence and absence of the pollutants was carried out by Fourier transform infrared spectrometer with attenuated total reflectance (ATR) attachment under dry air at room temperature (PerkinElmer, Spectrum Two, Singapore). Sample aliquots were centrifuged (8000*g*, 10 min) followed by washing with distilled water and the pellets obtained were vacuum dried prior to FTIR analysis. The samples were uniformly mixed with KBr in 100:1 ratio. The analysis was performed over the entire wave number range with 20 consecutive scans at a 4.0 cm^−1^ resolution. FTIR spectra were taken under the transmittance mode.

For FESEM–EDX analysis, biomass samples were vacuum dried and mounted on aluminum stubs over double-sided carbon tape followed by double coating with thin gold layer by sputter coater.

## Results and discussion

### *R. opacus* biomass growth and anthracene biodegradation in the presence of heavy metals

**Fig. 1:**
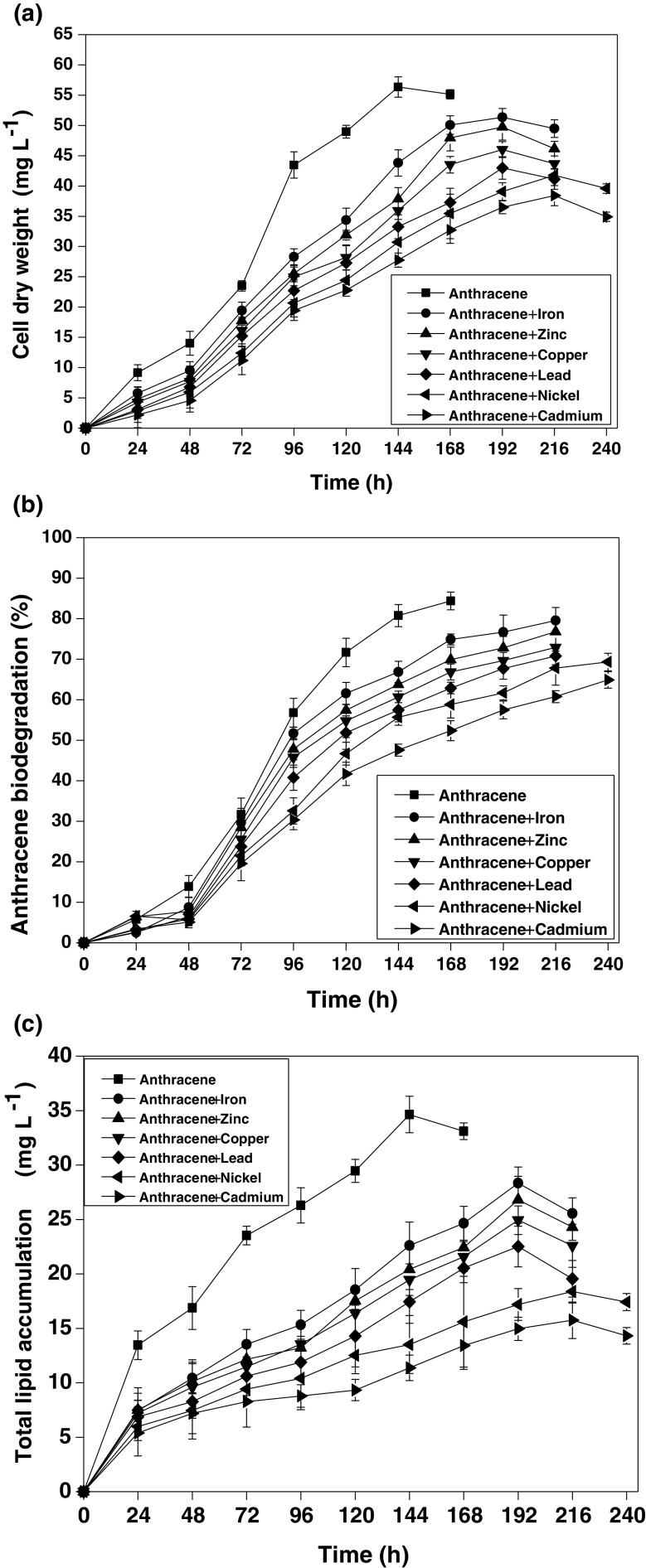
Time profile of **a** anthracene biodegradation; **b** biomass grown and **c** total lipid accumulation by *R. opacus* in the presence of different heavy metals (initial anthracene concentration = 100 mg L^−1^; initial heavy metal concentration = 10 mg L^−1^)

**Fig. 2:**
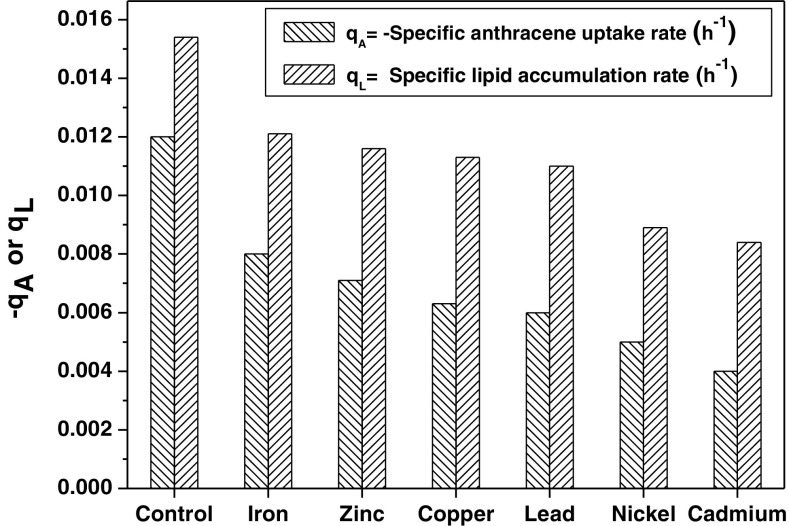
Estimated specific anthracene uptake rate and specific lipid accumulation rate by *R. opacus* (initial anthracene concentration = 100 mg L^−1^, initial heavy metal concentration = 10 mg L^−1^)

The capability of *R. opacus* to simultaneously biodegrade anthracene and accumulate lipids in the presence of different heavy metals was studied and is shown in Fig. 1a–c. These results reveal that in the presence of heavy metals, the bacterium showed a reduced anthracene biodegradation rate and efficiency along with a decrease in both biomass and total lipid accumulation. A separate set of flasks without the inoculum served as the control in the experiment, which indicated that the abiotic loss of the PAH was within the range 1.6–4.7%. Figure 1a reveals a clear difference in the lag phase of biomass growth in the presence and absence of the heavy metals. The anthracene biodegradation efficiency decreased sharply in the presence of the heavy metals when compared to that of the control (Fig. 1b). The total time for maximum biomass growth also increased from 168 to 216 h in the presence of Fe, Cu, Zn, and Pb and to 240 h in the presence of Cd and Ni. The heavy metals effect on *R. opacus* biomass growth, lipid accumulation, and anthracene biodegradation followed the order: Cd > Ni > Pb > Cu > Zn > Fe. A sharp decline in anthracene biodegradation efficiency in the presence of the heavy metals is observed after 72–84 h, which correlates well with the decline in the cell dry weight of the bacterium. Figure 2 further shows that a decrease in both specific anthracene consumption rate (−*q*_A_) and lipid accumulation rate (*q*_L_) is observed in the presence of these heavy metals.

A similar trend in biomass growth and total lipid content in the presence of the heavy metals is observed for the other initial anthracene concentration (100 mg L^−1^) (data not shown). These results suggested that the heavy metals are primarily responsible for inhibition of key enzymes involved in anthracene uptake, biomass growth, and lipid accumulation by *R. opacus* (Maria et al. 2013). The effect due to heavy metals on biomass growth, anthracene biodegradation, and lipid accumulation by *R. opacus* correlates well with the observation that the residual concentration of heavy metals at 24 h of the experiments is found to be nil (data not shown). The heavy metal removal mechanism by the organism is primarily attributed to their quick biosorption followed by their uptake inside the cells, which is consistent with literature reports that microbial biosorption significantly influences the biodegradation of recalcitrant compounds (Roy et al. 2015; Lu and Zhu 2012; Sokhn et al. 2001). Bueno et al. (2008) investigated the sorption capacity of non-viable *R. opacus* to bind with heavy metals (Pb, Cu, and Cr) from aqueous environment and observed that the sorption phenomena followed the pseudo-second order kinetics. In the literature, Chen et al. (2013) also reported that Cu(II) inhibited benzo [a] pyrene biodegradation by *Stenotrophomonas maltophilia* owing to the bacterial cell wall damage.

## Characterization of the bacterial biomass

### FESEM, EDX, and TEM analyses

**Fig. 3:**
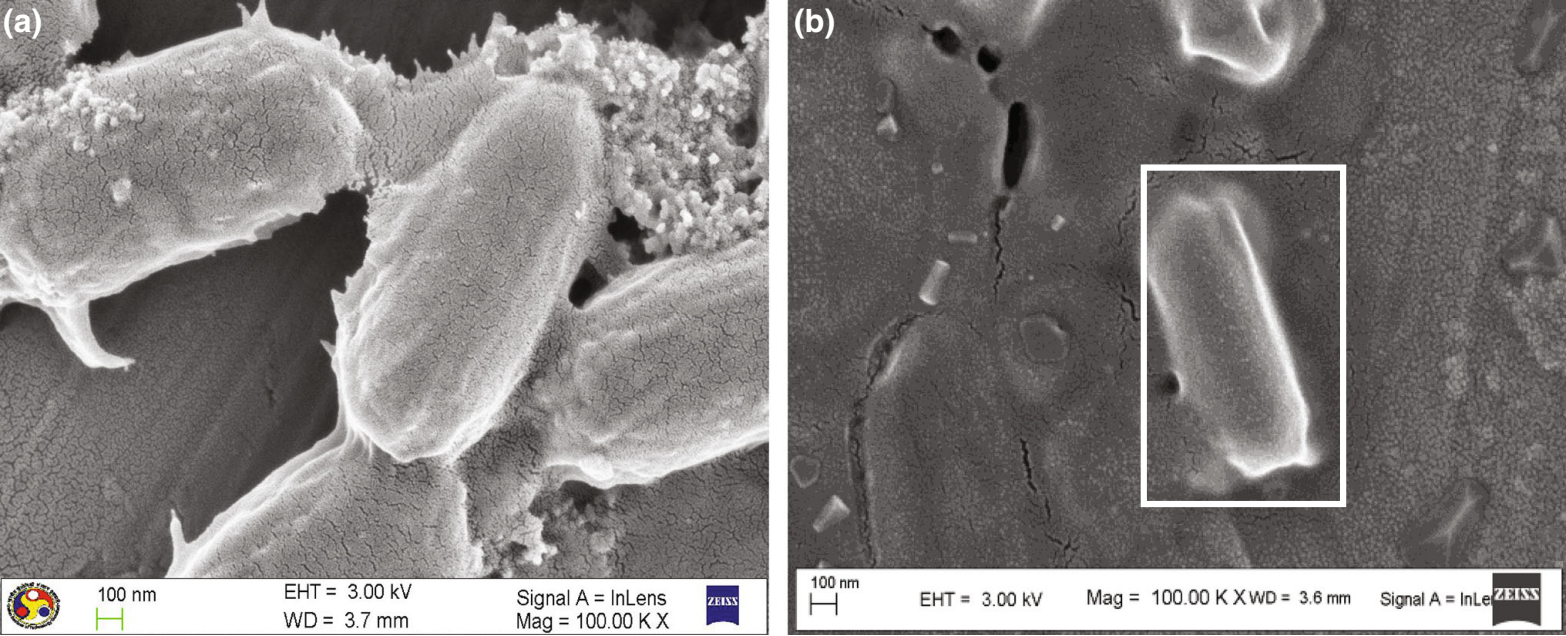
FESEM image of the bacteria **a** control biomass and **b** heavy metal loaded biomass

**Fig. 4:**
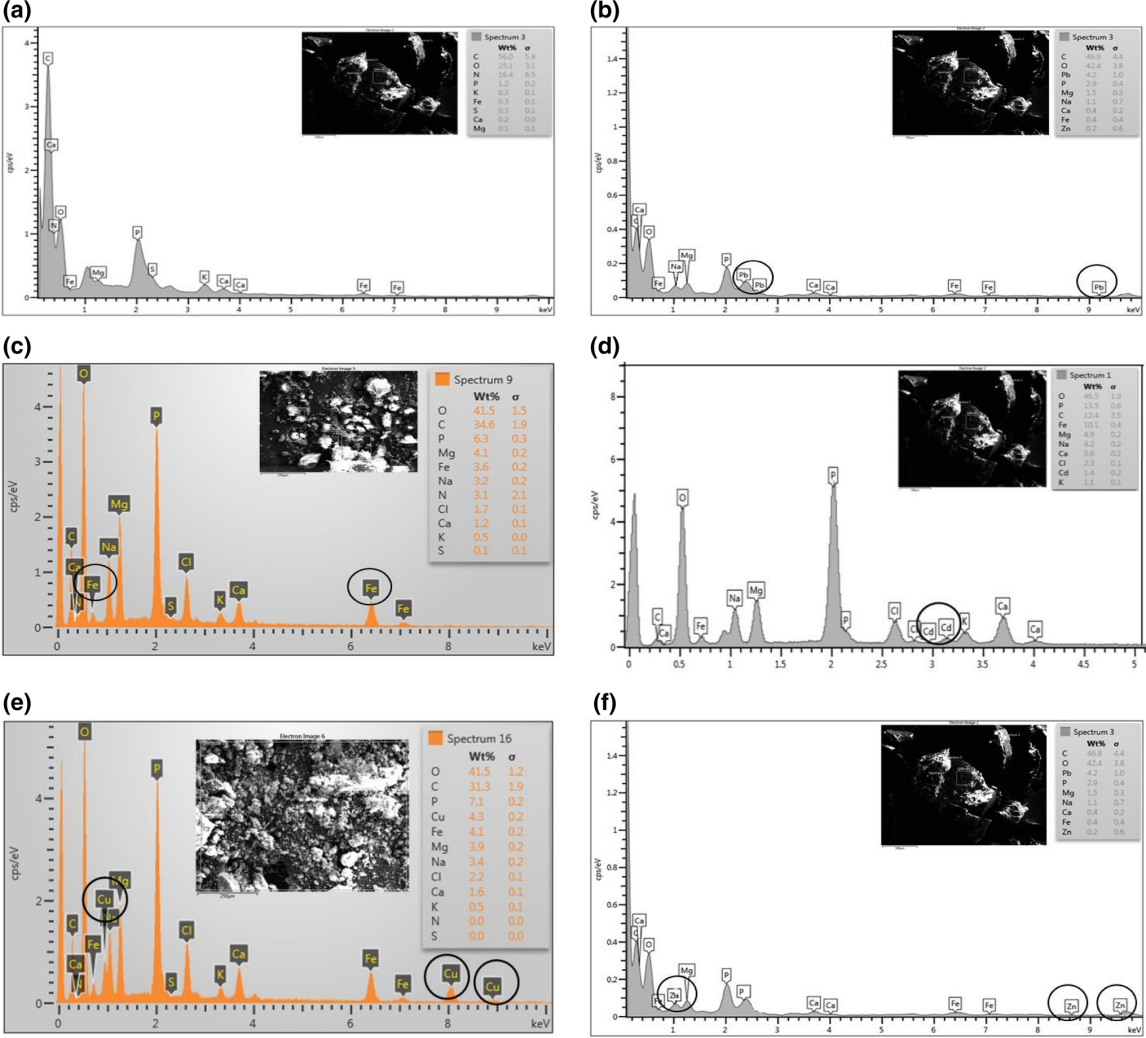
EDX spectrum of **a** control biomass and **b** biomass loaded with Pb(II); **c** Fe(III); **d** Cd(II); **e** Cu(II); **f** Zn(II).* Insert* shows the FESEM image of the biomass loaded with the respective metals

**Fig. 5:**
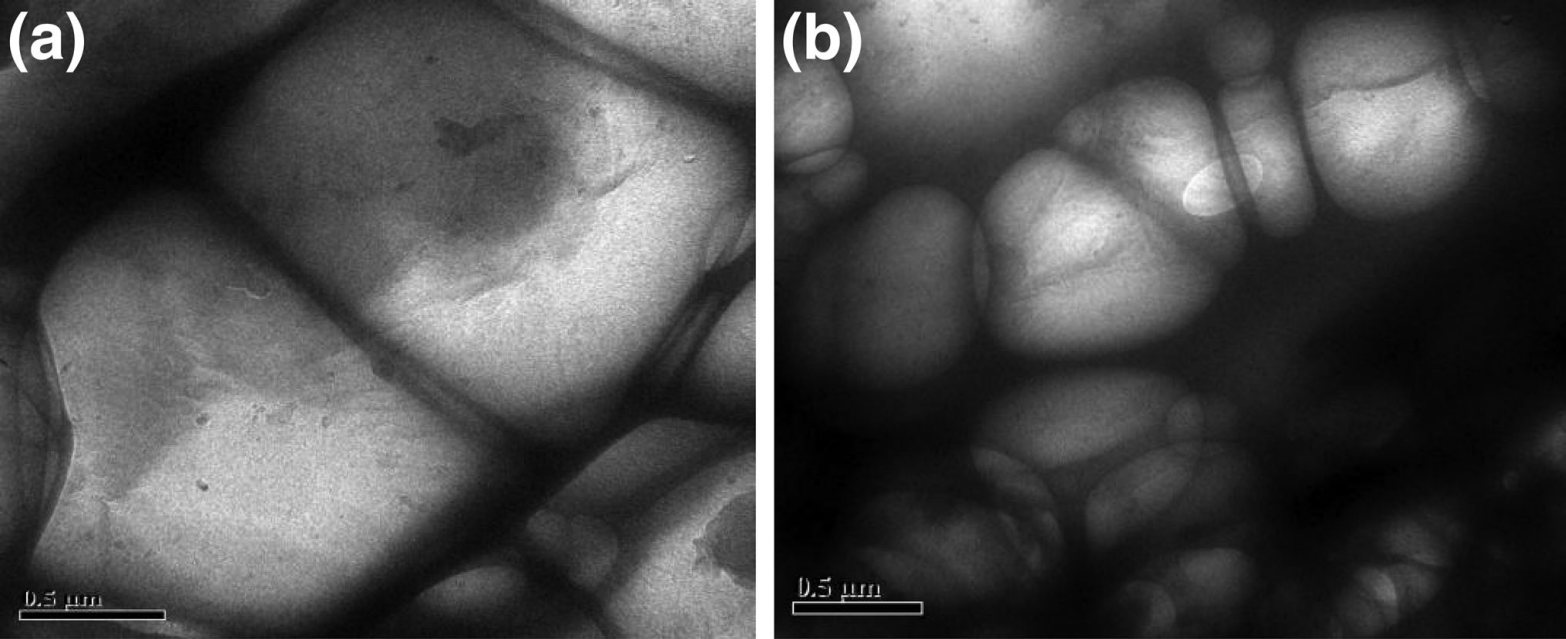
TEM image of lipid globules accumulated inside the bacteria: (**a**) control biomass and (**b**) heavy metal loaded biomass

For understanding the changes in morphology and elemental composition of the *R. opacus* biomass grown in presence of the heavy metals, FESEM–EDX analysis of control biomass and the metal loaded biomass was performed. Figure 3a, b clearly shows morphological difference in the bacterial cell due to the heavy metals. Figure 4a–f displays the EDX spectrum of the control and the heavy metal loaded biomass, which clearly reveals an extra peak due to the respective heavy metals on the biomass. These results confirmed that heavy metal uptake by the bacteria is due to biosorption. The inhibitory effect of the heavy metals on lipid accumulation by the bacterium is evident from the small-sized globules of the biomass observed under TEM (Fig. 5a, b). Whereas Fig. 5a shows TEM image of control biomass grown without any heavy metals, Fig. 5b shows TEM image of biomass grown in the presence of heavy metals.

### FTIR spectroscopy analysis

**Fig. 6:**
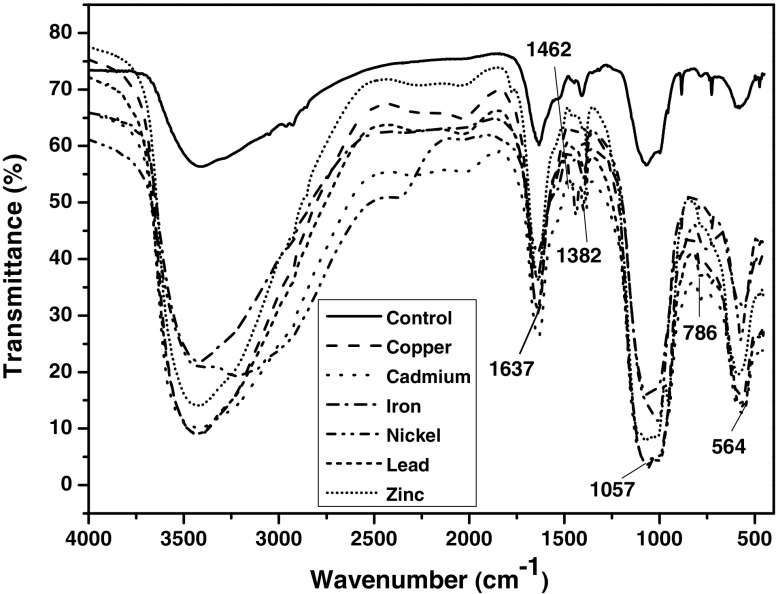
FTIR spectra of control biomass and metal loaded biomass

The bacterial biomass obtained from the experiments was further characterized utilizing Fourier transform infrared (FTIR) spectrometer to identify the interaction between the heavy metal ions and the functional group involved on the bacterial surface (Fig. 6). The control biomass depicted strong peaks at 1637, 1462, 1382, 1057, 786, and 564 cm^−1^, which are attributed to primary amine of proteins (amide III, –N–H) stretch (1650–1580 cm^−1^), C–H alkane bend (1470–1450 cm^−1^), –CH_2_–(C=O) stretch (1400–1370 cm^−1^), secondary amide γ(N–H) + v(C–N), –SO_3_H stretch, pyridine (I) β(C–H) and pyridine (II) β(C–H) (1300–850 cm^−1^), C–H and O–H bending stretch (750–700 cm^−1^), and alkyl halide (–C–Cl) stretch (850–550 cm^−1^), respectively (Ye et al. 2013). Comparison of the spectra due to control biomass with that of the heavy metal loaded biomass reveals that N–H, C–H bend, –CH_2_–(C=O), C–N stretch, C–H and O–H bending, and –C–Cl stretch participated in heavy metal binding by the bacterium. These FTIR results confirm the involvement of peptidoglycan present in the bacterial cell wall for heavy metal binding (Wei et al. 2011).

## Conclusions

This study demonstrated simultaneous heavy metal removal and anthracene biodegradation by *Rhodococcus opacus*. However, heavy metal biosorption by the bacterium negatively influences its biomass growth, anthracene biodegradation, and total lipid accumulation. The heavy metal effect followed the order: Cd > Ni > Pb > Cu > Zn > Fe. The qualitative assessment of negative effect imposed by heavy metals on the biomass surface and lipid accumulation was successfully characterized using FESEM and TEM analyses. FESEM–EDX analysis of the bacteria biomass further confirmed that the metal precipitates formed were associated with the cell surface. FTIR characterization of the biomass grown in the absence and presence of the metals further confirmed the involvement of N–H, C–H bend, –CH_2_–(C=O), C–N stretch, C–H and O–H bending, and –C–Cl groups on the biomass for heavy metal binding by the bacteria. Overall, this study proved the potential of the strain to remove both organic and inorganic pollutants from mixture as well as accumulate lipid inside by utilizing such recalcitrant pollutants. The accumulated lipids can further be transesterified for its potential biodiesel application.
